# Efficacy of sacubitril/valsartan combined with dapagliflozin in treating patients with heart failure and diabetes after an acute myocardial infarction

**DOI:** 10.12669/pjms.40.11.8972

**Published:** 2024-12

**Authors:** Lifang Zhang, Xiuying Tang, Runjun Li, Jinxia Niu, Jie Gong

**Affiliations:** 1 Lifang Zhang Comprehensive Internal Medicine The First Hospital of Qinhuangdao, Qinhuangdao 066000, Hebei, China; 2 Xiuying Tang Department of Cardiology The First Hospital of Qinhuangdao, Qinhuangdao 066000, Hebei, China; 3 Runjun Li Department of Critical Care Medicine People’s Hospital of Yangjiang, Yangjiang 529599, Guangdong, China; 4 Jinxia Niu Department of Cardiology The First Hospital of Qinhuangdao, Qinhuangdao 066000, Hebei, China; 5 Jie Gong Department of Critical Care Medicine People’s Hospital of Yangjiang, Yangjiang 529599, Guangdong, China

**Keywords:** Acute myocardial infarction, Heart failure, Diabetes, Sacubitril/valsartan, Dapagliflozin, Therapeutic effect

## Abstract

**Objective:**

To investigate the efficacy of sacubitril/valsartan combined with dapagliflozin in diabetic patients suffering from heart failure following an acute myocardial infarction.

**Methods:**

This retrospective study included 80 diabetic patients who had heart failure after an acute myocardial infarction (AMI) and were hospitalised at First Hospital of Qinhuangdao between January 2021 and January 2023. They were randomly divided into the control and observation groups (each group n = 40). In addition to fundamental anti-heart failure treatment, the control group was administered with sacubitril/valsartan, whereas the observation group received sacubitril/valsartan combined with dapagliflozin. The treatment lasted for six months, and the patient’s glucose-associated targets and prognosis before and after treatment were compared in both groups.

**Results:**

Six months after the treatment, the fasting blood glucose, 2-h postprandial blood glucose and HbA1c levels of patients from both groups significantly decreased. The decrease was more prominent in the observation group than in the control group, with significant differences (*P <* 0.05). Additionally, the LVEF, CO, CI and NT-proBNP levels considerably decreased in both groups after treatment, with the observation group exhibiting a more notable decrease that was significantly different (*P <* 0.05). An inter-group comparison showed that the differences were not significant (*P >* 0.05).

**Conclusions:**

In diabetic patients suffering from heart failure after an AMI, treatment with sacubitril/valsartan combined with dapagliflozin may effectively control blood glucose levels and improve cardiac functions without increasing adverse reactions, indicating high clinical safety.

## INTRODUCTION

Acute myocardial infarction (AMI) is a common severe disease. Acute ischaemia and cardiac muscle tissue necrosis are caused by a dramatic decrease in or interruption of blood supply by coronary arteries. AMI can lead to abnormalities in ventricular contractions and ventricular diastole, which further induces heart failure (HF).^1^,^2^ AMI morbidity increases annually with improvements in living standards, changes in dietary habits and impacts of population ageing and psychological factors.

Relevant data shows that approximately 56% of AMI patients exhibit cardiac function diminution, and 1/3 of them develop HF.^3^,^4^ According to statistics, patients with both AMI and acute HF have an in-hospital mortality rate of approximately 3% and a half-year rehospitalisation rate of about 5%.^5^ Moreover, up to 60% of these patients may die in five years. Diabetes mellitus (DM), especially type-2 DM (T2DM), is a common condition. As revealed in relevant data,^4^ the probability for patients with both T2DM and HF to suffer MACE is 2–3 times greater than that for T2DM patients with no HF. In typical cases, HF patients show abnormalities in insulin and glucose metabolism. Clinical data shows that patients who suffer from HF and T2DM following AMI are characterised by high treatment difficulties, high mortality and poor prognosis.^6^

In such patients, glucose control conditions should be highlighted during HF treatment to radically resolve HF symptoms. Sacubitril/valsartan is a common drug in HF treatment and can suppress angiotensin receptors, realise vasodilation, improve cardiovascular functions and relieve HF symptoms. Additionally, dapagliflozin is a novel blood glucose control drug. It blocks urine sodium reabsorption, reduces blood glucose and protects the cardiovascular system by suppressing sodium–glucose cotransporter2 (SGLT2). Currently, clinical applications of sacubitril/valsartan combined with dapagliflozin in the treatment of T2DM patients who suffer from HF following AMI are rare. Thus, this study aimed to investigate the clinical effects of combining sacubitril/valsartan with dapagliflozin in the treatment of T2DM patients who develop HF after AMI.

## METHODS

This was a retrospective study. Eighty HF patients with T2DM who suffered from AMI hospitalised at First Hospital of Qinhuangdao between January 2021 and January 2023 were prospectively selected. Using the random number table, they were divided into the observation group administered with sacubitril/valsartan combined with dapagliflozin and the control group treated with sacubitril/valsartan only; each group included 40 patients.

### Ethical Approval:

The study was approved by the Institutional Ethics Committee of First Hospital of Qinhuangdao (Ref. No. 2022A019; dated March 3, 2022). Written informed consent was obtained from all participants.

### Inclusion criteria:

- Cardiac functions at classes II–IV according to the New York Heart Association Classification of Heart Failure^7^,^8^
- Conforming to HF and T2DM diagnosis standards after suffering from AMI
- Left ventricular ejection fraction (LVEF) <40%
- No medical history of HF
- No contraindications for sacubitril/valsartan and dapagliflozin and no history of use of these drugs
- Signed corresponding informed consent

### Exclusion criteria:

- Medical history of HF
- Allergy to or contraindications for sacubitril/valsartan and dapagliflozin
- Malignant arrhythmia or myocardial diseases
- Recurrent hypoglycaemia, hypotension and malignant tumours
- Diabetes and kidney diseases

### Therapeutic methods:

The patients with close restraints over water and sodium intake received basic treatment and underwent electrocardiograph monitoring and oxygen inhalation. Routine blood glucose reduction and anti-HF treatment were performed. Patients in the control group were treated by oral administration of sacubitril/valsartan (dosage: 100 mg twice a day). In the observation group, sacubitril/valsartan was administered in combination with dapagliflozin at a dose of 10 mg once a day. The medication was taken orally with an empty stomach in the morning. Respective therapies continued for six months in each group.

### Observation targets:

- Glucose-associated indicators: Fasting blood glucose, two hours postprandial blood glucose (2hPBG) and haemoglobin A1c (HbA1c) levels before and six months after the treatment were compared among patients in both groups.
- Cardiac function indicators: Colour Doppler ultrasound examinations were performed before and six months after the treatment to measure and compare LVEF, cardiac output (CO) and cardiac indexes among patients in both groups.
- Serological indicators: N-terminal pro-brain natriuretic peptide (NT-proBNP) serum levels before and six months after the treatment were compared for patients in both groups.
- Clinical safety: The occurrence rates of adverse reactions during the treatment of patients in both groups were compared, including angioedema, hypotension, hyperglycaemia, hypoglycaemia and renal function impairment.
- Prognosis comparison: After the participants were discharged from the hospital, follow-up visits were conducted for six months. Every four weeks, they were followed up over the telephone or in the outpatient department. In cases of any discomfort, the participants were instructed to consult medical advice at any time. During follow-up, the rehospitalisation and occurrence rates of MACE in patients in both groups were recorded.

### Statistical Analysis:

Statistical analyses were performed using SPSS 21.0. Quantitative data are denoted by mean ± standard deviation (*χ̅* ± *S*) and an independent sample t-test for inter-group comparison. A 95% confidence interval was used; qualitative data were represented as the number of cases and their percentages [n(%)], and c^2^ test was conducted for corresponding inter-group comparison. *P <* 0.05 indicated a significant difference.

## RESULTS

The observation group consisted of 25 male and 15 female patients aged 40–70 (mean age: 40–70). Conversely, the control group included 23 male and 17 female patients aged 40–68 (mean age: 56.08 ± 8.98). The general data of patients in both groups showed no significant differences (*P >* 0.05) but were comparable (Table I).

**Table-I:** Comparison of general data between the observation and control groups.

Items	Observation (n=40)	Control (n=40)	t/c^2^	P
Age	56.23±10.64	55.83±8.83	0.183	0.855
Sex (M/F)	25/15	23/17	0.208	0.648
Duration of diabetes (years)	6.63±1.58	6.93±1.64	0.833	0.407
NYHA grading			0.077	0.962
II	14 (12.50%)	13 (10.00%)		
III	12 (17.50%)	13 (17.50%)		
IV	14 (7.50%)	14 (5.00%)		
***History of drug therapies***				
Sulfonylureas	38	36	0.721	0.396
Ndinedglinide	32	35	0.827	0.363
Biguanides	36	31	2.296	0.130
Glitazones	33	36	0.949	0.330
Insulin	30	33	0.672	0.412

No statistical significance was found in the FPG, 2hPBG and HbA1c levels of patients in both groups before treatment. However, the FPG, 2hPBG and HbA1c levels dramatically decreased after treatment and were more prominent in the observation group. The differences before and after treatment were significant (*P <* 0.05, Table II).

**Table-II:** Blood glucose comparison before and after treatment of patients in the observation and control groups (*χ̅*±*S*).

Groups	FBG		2hPBG		HbA1c	

	Before treatment	After treatment	Before treatment	After treatment	Before treatment	After treatment
Observation (n=40)	10.04±3.17	5.87±1.77	14.68±3.86	8.11±1.97	9.49±0.66	6.15±0.41
Control (n=40)	10.04±2.73	7.08±2.12	14.92±2.55	9.53±2.04	9.51±0.75	7.55±0.76
*t-value*	0.011	2.758	0.332	3.168	0.091	10.295
*P-value*	0.991	0.007	0.741	0.002	0.928	0.000

No statistical significance was noted in LVEF, CO and CI of patients in both groups before treatment (*P >* 0.05). Three months after treatment, LVEF, CO and CI levels considerably increased in both groups; the increase was higher in the observation group than in the control group. The differences were found to be significant (*P <* 0.05, Table III).

**Table-III:** Comparison of cardiac function indicators before and after treatment of patients in the observation and control groups (*χ̅*±*S*).

Groups	LVEF (%)		CO(L·min)		CI (L·min·m^2^)	

	Before treatment	After treatment	Before treatment	After treatment	Before treatment	After treatment
Observation (n=40)	33.31±4.12	48.57±6.37	3.19±0.10	4.96±0.18	2.75±0.27	3.73±0.26
Control (n=40)	33.52±3.83	45.59±6.14	3.15±0.13	4.86±0.16	2.72±0.31	3.59±0.29
*t-value*	0.236	2.132	1.641	2.418	0.457	2.273
*P-value*	0.814	0.036	0.105	0.018	0.649	0.026

NT-proBNP was not significantly different between patients in both groups before treatment (*P >* 0.05). Six months following treatment, NT-proBNP levels in patients from both groups were considerably reduced compared to those before the treatment; the decrease was more prominent in the observation group. Their differences were significant (*P <* 0.05, Table IV).

**Table-IV:** NT-proBNP comparison before and after treatment of patients in the observation and control groups (*χ̅*±*S*).

	Observation (n=40)	Control (n=40)	t-value	P-value
Before treatment	10149.61±850.87	10104.57±864.21	0.235	0.815
After treatment	195.20±16.39	351.31±20.76	37.327	0.000
*t-value*	73.978	71.356		
*P-value*	0.000	0.000		

During treatment, adverse reactions were observed among patients of both groups. However, occurrence rates of such adverse reactions were comparatively low, showing no significant differences (*P >* 0.05, Table V).

**Table-V:** Comparison of adverse reactions during the treatment of patients in the observation and control groups [Case number (%)].

Groups	Angio-edema	Hypotension	Hyperglycemia	Hypoglycemia	Renal function impairment	Overall response rate
Observation (n=40)	1 (2.50)	1 (2.50)	1 (2.50)	1 (2.50)	1 (2.50)	5 (12.50)
Control (n=40)	1 (2.50)	0 (0.00)	1 (2.50)	1 (2.50)	1 (2.50)	4 (10.00)
*c* ^2^ *-value*						0.125
*P-value*						0.723

At follow-up, the rehospitalisation and MACE occurrence rates were 2.50% (1/40) and 2.50% (1/40), respectively, in the observation group and 15.00% (6/40) and 17.50% (7/40), respectively, in the control group. Moreover, inter-group differences were significant (*P <* 0.05, Table VI).

**Table-VI:** Prognosis comparison among patients in the observation and control groups [Case number (%)].

Groups	Rehospitalization rates	MACE occurrence rates
Observation (n=40)	1 (2.50)	1 (2.50)
Control (n=40)	6 (15.00)	7 (17.50)
c^2^-value	3.914	5.000
P-value	0.048	0.025

## DISCUSSIONS

The higher the NT-proBNP concentration, the greater the risk of sudden death from HF. This study indicated that cardiac function indicators (e.g. LVEF, CO and CI levels and NT-proBNP concentrations) significantly improved in patients after the treatment and were more significant among patients in the observation group (*P* < 0.05). This shows that combining sacubitril/valsartan with dapagliflozin can significantly improve the cardiac functions of T2DM patients suffering from HF after AMI.

FPG and 2hPBG are indicators commonly used for glucose monitoring in clinics. HbA1c is beneficial in evaluating glucose control effects. In cases of poor glucose control, levels of FPG, 2hPBG and HbA1c are all above normal values. Glucose control is critical to slow down disease progression in T2DM patients suffering from HF after AMI. However, some hypoglycaemic agents other than dapagliflozin may induce HF among diabetes patients. In the present study, the FPG, 2hPBG and HbA1c levels considerably reduced in both groups six months after treatment, and the decline was more prominent in the observation group (*P* < 0.05).

The study results showed that the combined use of sacubitril/valsartan and dapagliflozin can significantly reduce blood glucose levels in T2DM patients suffering from HF after AMI, establishing a foundation for regulating patients’ ventricular ejection fractions. Regarding occurrence rates of adverse reactions in both groups, no statistical significance was found in the present study. The rehospitalisation and MACE occurrence rates were lower in the observation group than in the control group, and their differences were significant (*P* < 0.05). This indicates that the use of sacubitril/valsartan combined with dapagliflozin can lower rehospitalisation and MACE occurrence rates of patients without prominent increases in their adverse reactions, indicating a safe and reliable clinic application.

For patients suffering from AMI, acute coronary occlusion and blood flow interruption lead to ischaemic and hypoxic necrosis of myocardial cells, which is mainly manifested by intense and persistent substernal pain and myocardial enzyme increase.^9^ Most AMI patients also suffer from HF. In the existing literature,^10^ the morbidity of AMI occurring along with HF is approximately 32.4%. Furthermore, long-term hyperglycaemia in T2DM patients can induce oxidative stress and inflammatory responses, impair vascular endothelial cells and cause rheological blood changes and microcirculation dysfunction. Consequently, myocardial ischaemia and anoxia become more severe in AMI patients, leading to cardiac function deterioration. Relevant research showed that T2DM patients are more likely to suffer from HF as the onset of AMI and their mortality are relatively high.^11^,^12^ Considering this, T2DM patients suffering from HF after AMI are actively and effectively treated in clinics to avoid rehospitalisation and death, extend survival time and improve prognosis.

Currently, routine symptomatic and supportive therapies are generally selected for T2DM patients suffering from HF following AMI, such as oxygen inhalation, glucose reduction, diuresis inducing and cardiac load decreasing. Routine treatment is effective in controlling clinical symptoms; however, it induces poor long-term efficacy. Sacubitril/valsartan is a novel anti-heart failure drug^13^ composed of sacubitril and valsartan. Under actions of liver fusoacetase, sacubitril is decomposed into enkephalin inhibitors that suppress the effects of neprilysin, blocking endogenous natriuretic peptide degradation, raising natriuretic peptide concentration, regulating vasoconstriction, lowering anterior and posterior cardiac loads and inhibiting ventricular remodelling. This relieves symptoms of HF. Valsartan angiotensin II receptor binds with type II-1 receptor to treat HF. Dapagliflozin has been new to the market in recent years.^14^ As an SGLT2 inhibitor, it brings down renal glucose thresholds by inhibiting Na^+^ and glucose reabsorption by Na^+^SGLT-2 in the proximal convoluted tubule to facilitate glucose excretion to the outside of the body through urine. Additionally, it protects pancreatic beta cell functions and effectively reduces HbA1c to regulate and control blood glucose levels.

Furthermore, dapagliflozin can suppress the overactivation of renin–angiotensin and sympathetic nervous systems and inhibit the transformation of glucose into fatty acid oxidation, which is beneficial for glucose regulation and control in T2DM patients. Several clinical trials have demonstrated that using both sacubitril/valsartan and dapagliflozin can boost cardiac functions.^15^-^17^ With respect to NT-proBNP, it is a heart hormone that regulates cardiovascular stability. In clinics, it is frequently used as a biomarker of HF.^18^

### Limitations:

It includes a small sample size. Further investigations should be performed using a larger sample size.

## CONCLUSIONS

Use of sacubitril/valsartan combined with dapagliflozin in treating T2DM patients suffering from HF after AMI may control blood glucose, improve cardiac functions and reduce rehospitalisation and MACE occurrence rates without increasing adverse reactions. Such a regimen is feasible and safe to use in clinics.

### Author's Contribution:

- - - - - - - - - - - - - - - - - - - - - - - - - - - - - - - - - - - - - - - - - - - - - -

- - - - - - - - - - - - - - - - - - - - - - - - - - - - - - - - - - - - - - - - - - - - - -

- - - - - - - - - - - - - - - - - - - - - - - - - - - - - - - - - - - - - - - - - - - - - -

- - - - - - - - - - - - - - - - - - - - - - - - - - - - - - - - - - - - - - - - - - - - -

- - - - - - - - - - - - - - - - - - - - - - - - - - - - - - - - - - - - - - - - - - - - -.
